# Supplementary material for: Self-Supported 3D PtPdCu Nanowires Networks for Superior Glucose Electro-Oxidation Performance
Source: Molecules. 2023 Aug 2;28(15):5834. doi: 10.3390/molecules28155834 (PMC10421379; doi:10.3390/molecules28155834)
Supplement: Supplementary file 1 [file molecules-28-05834-s001.zip › molecules-2411888-supplementary.pdf]

Supplementary Materials

Self-Supported 3D PtPdCu Nanowire Networks for Superior Glucose Electro-Oxidation Performance

Kaili Wang 1,2,3†, Shuang He 4†, Bowen Zhang 1, Zhen Cao 2, Tingting Zhou 2, Jia He 3* and Ganghui Chu 1*

[1] Laboratory of Xinjiang Native Medicinal and Edible Plant Resources Chemistry, Kashi University, Xinjiang, China

[2] College Chemistry & Chemistry Engineering, Weifang University, Shandong, China

[3] School of Materials Science and Engineering, Tianjin University of Technology, Tianjin, China

[4] First Teaching Hospital of Tianjin University of Traditional Chinese Medicine, Tianjin, China.

[*] Correspondence: cghks5@126.com; hejia@tjut.edu.cn

[†] These authors contribute equally.

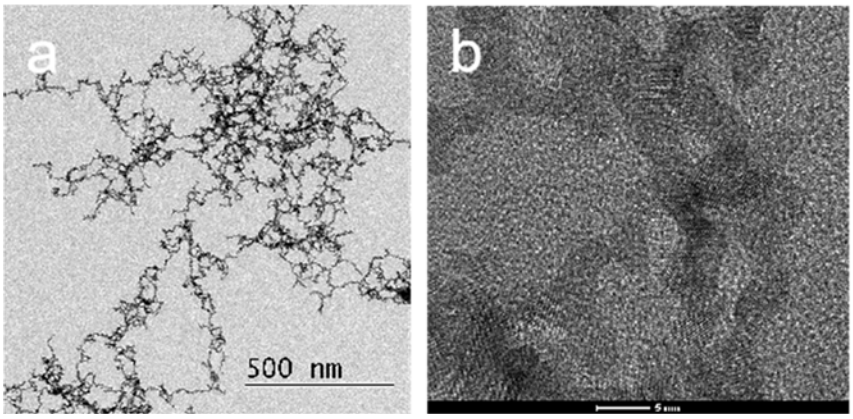

Figure S1. (a) TEM image of PtPdCu NWs, (b) HRTEM image of PtPdCu NWs.

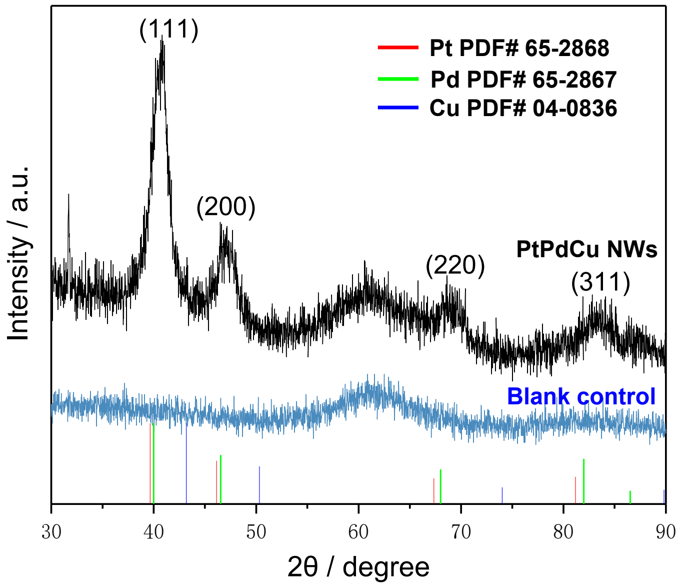

Figure S2. XRD pattern of PtPdCu NWs.

Z	Element	Family	Atomic Fraction (%)	Atomic Error (%)	Mass Fraction (%)	Mass Error (%)	Fit error (%)
29	Cu	K	26.32	3.51	14.06	1.32	0.51
46	Pd	L	46.84	7.75	41.92	5.69	0.34
78	Pt	L	26.84	4.30	44.02	5.70	0.22

Figure S3. The Pt/Pd/Cu atomic ratio of the PtPdCu NWs catalysts was determined by STEM-EDS spectrum.

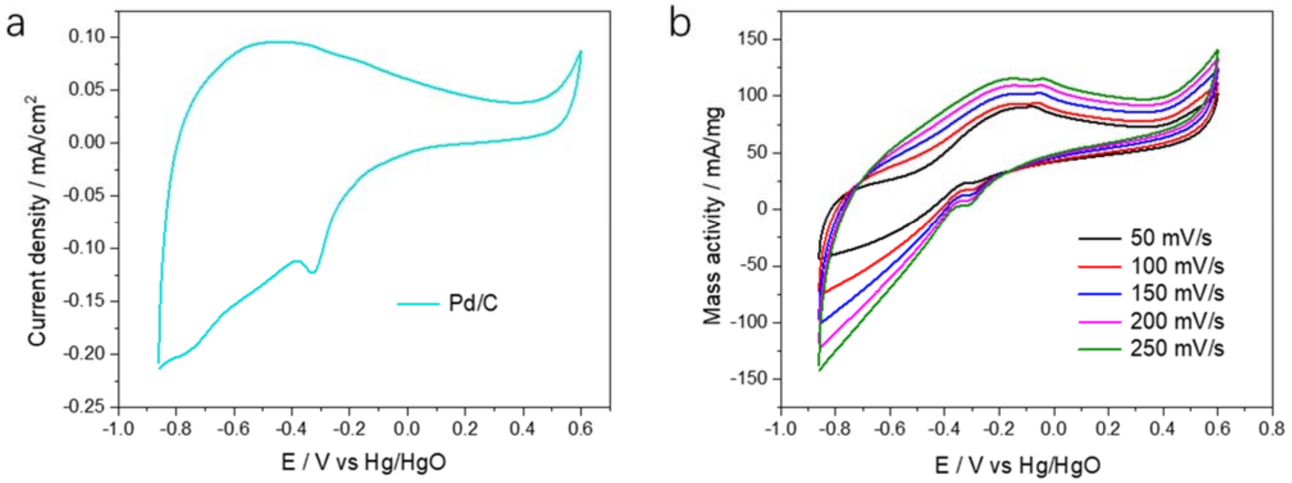

Figure S4. (a) CVs of the commercial Pd/C in 0.5 M KOH with scan rate of 50 mV/s; (b) CVs of the Pd/C in 0.5 M KOH containing 0.1 M glucose at different scan rates from 50 mV/s to 250 mV/s.

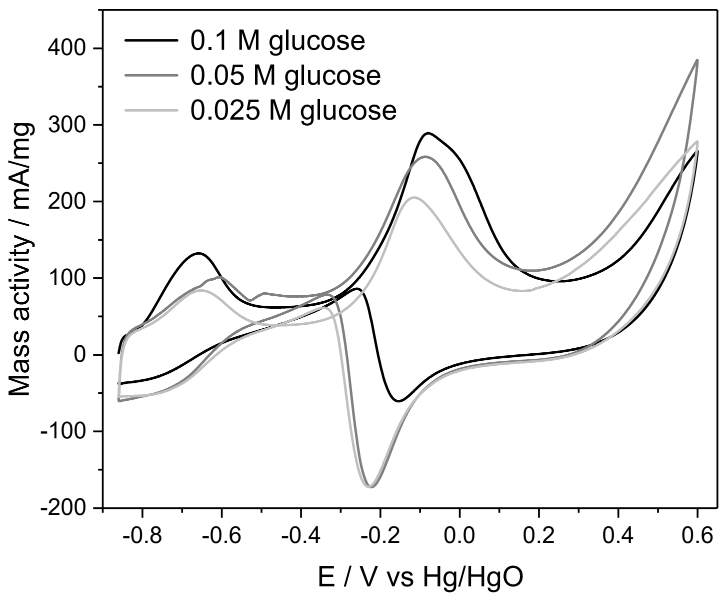

Figure S5. Glucose oxidation curves of the PtPdCu NWs catalyst in 0.1, 0.05, and 0.025 M glucose + 0.1 M KOH.

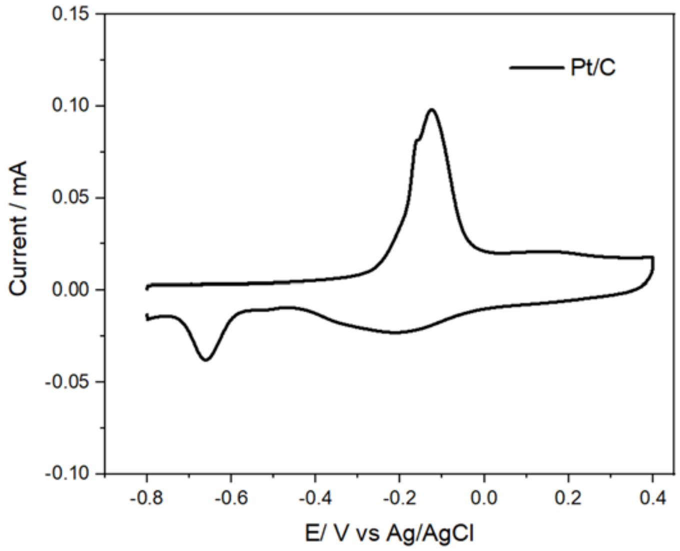

Figure S6. CO-stripping curves for Pt/C catalyst.

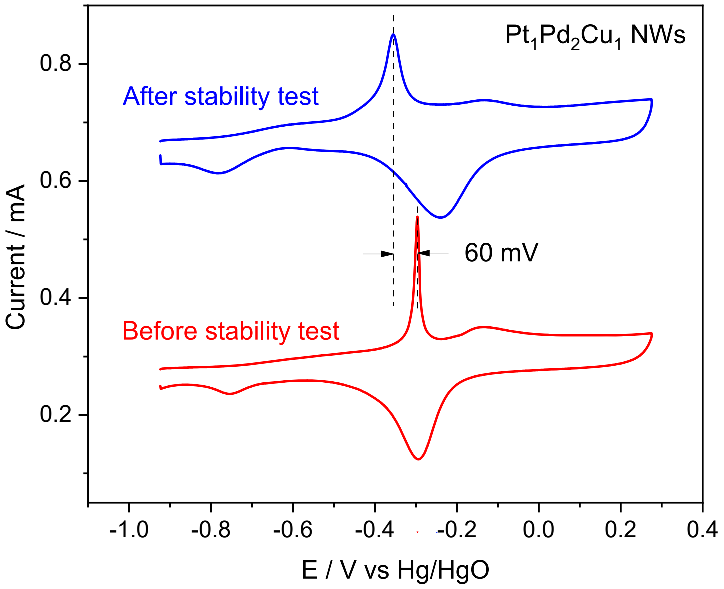

Figure S7. CO-stripping curves for $Pt_1Pd_2Cu_1$ NWs catalyst before and after the stability test.

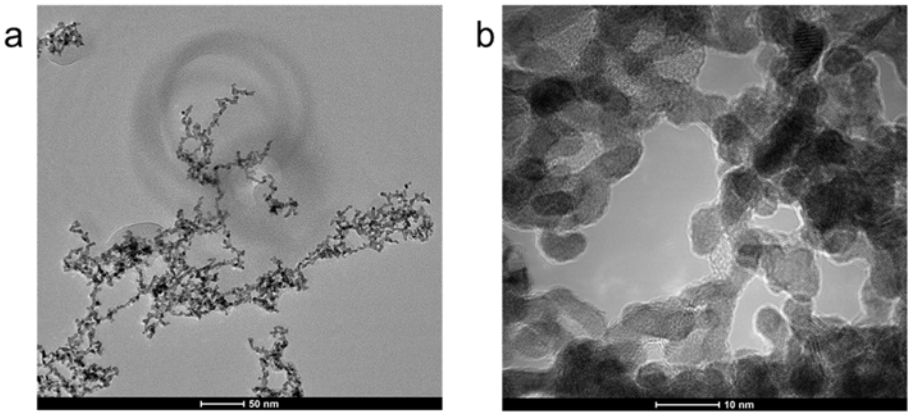

Figure S8. (a,b) TEM images of PtPdCu NWs catalysts after CA tests for GOR.

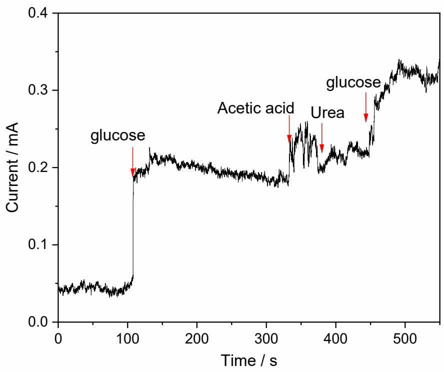

Figure S9. Amperometric response of the composite films by a consecutive addition of 1 mM of each of the indicated analytes.

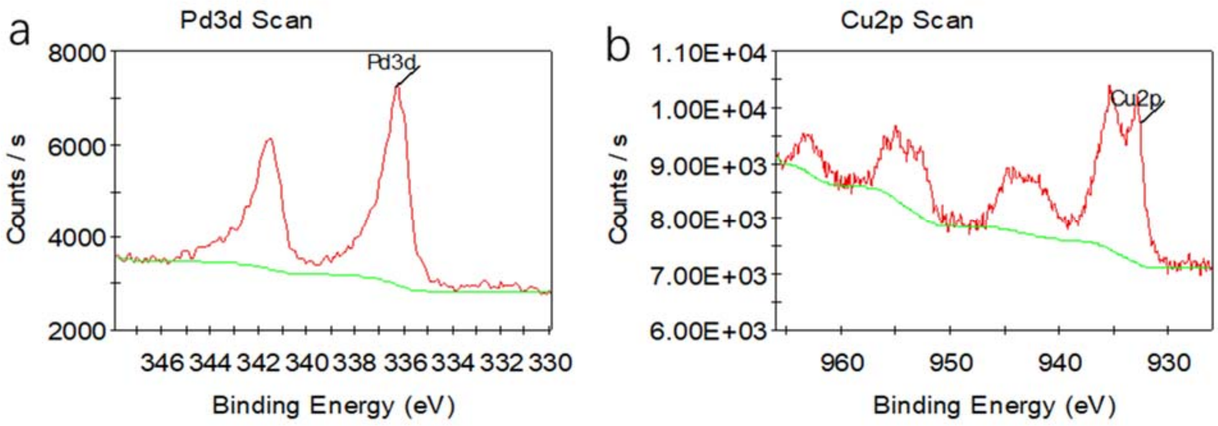

Figure S10. XPS patterns for (a) Pd 3d and (b) Cu 2p region of the as-prepared PtPdCu NWs.

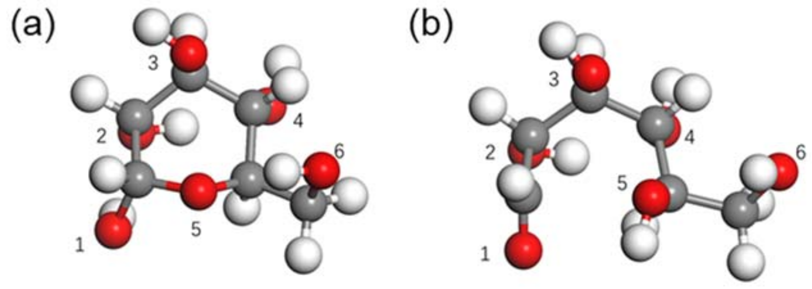

Figure S11. The two configurations of glucose molecules: (a) Ring and (b) Ring-first opened step.

In the Figure S11a, gray, red, and white spheres represent C, O, and H atoms, respectively. The sequence number represents the arrangement order of the O atoms. According to references [1, 2], the adsorption forms of glucose on the metal active sites Pd and Ni of two different materials are both at position 6, indicating that metals have greater adsorption potential on position 6th O, so the same adsorption site is used in this article.

In the Figure S11b, the reference information and order are the same as those described on the above. According to reference [3], the ring-opening mode of glucose molecular structure when it is not adsorbed on the surface is that the C atom bonded to the O atom at position 5 and the O atom at position 1 are broken. The bond O-H of the OH group at position 1 is broken, and the H atom moves to the O atom at position 5, thereby breaking the bond and opening the ring of the glucose molecule. At the beginning, when glucose was adsorbed on the surface, the surface of pure metal and ternary alloy materials strongly interacted with the active sites of 6th O atom. The active sites 1-5 were basically unaffected and the charge environment did not significantly change. Therefore, the same glucose ring opening method was selected. Due to the strong adsorption of metals on glucose molecules, after ring opening, the O site 6 still binds to the metal surface, but the original ring binding mode is destroyed. The O atom at position 5 loses the binding effect of the 1st O atom connecting the C atom and moves towards the 6th O atom and the metal surface, while the C atom connected to the 1st O atom loses the 5th binding force and extends away from the metal surface due to the molecular orbital hybridization force.

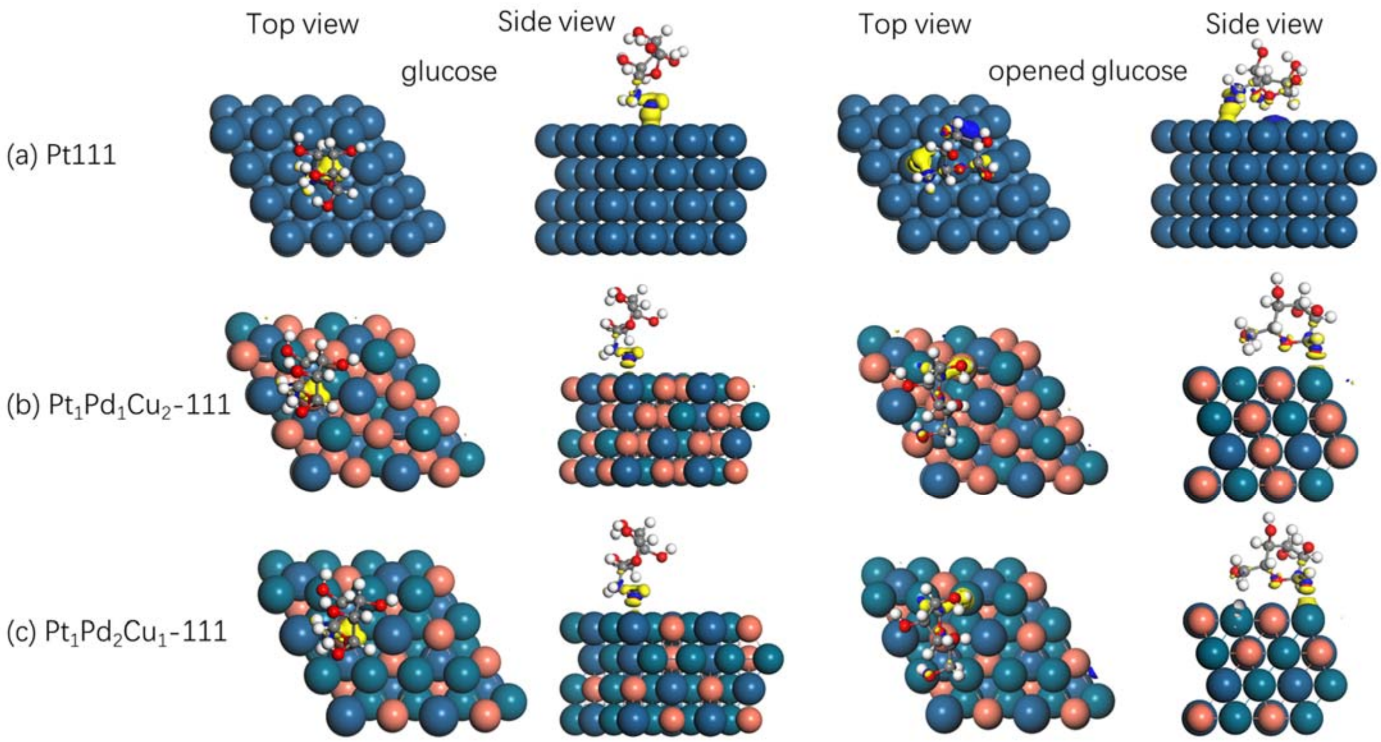

Figure S12. Electron density and Mulliken charge of (a) Pt111, (b) $Pt_1Pd_1Cu_2$-111 and (c) $Pt_1Pd_2Cu_1$-111. Two columns on the left represent glucose molecule adsorb on the three different models from the top and side views, and two columns on the right represent opened-glucose molecule adsorb on the three different models from the top and side views. The blue area in the figure represents electron accumulation, and the yellow area represents electron loss. Each arrow represents the direction of electron transfer, and the number next to the arrow represents the relative transfer quantity. Blue and red balls represent Pt and Cu atoms respectively, and the remaining balls are Pd atoms.

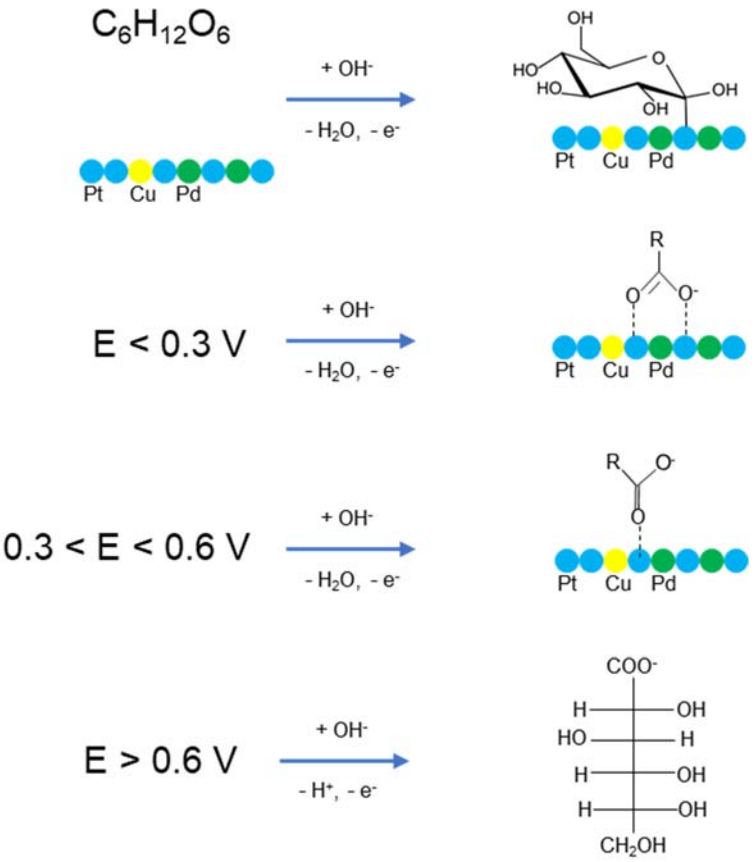

Figure S13. Reaction mechanism of the electrochemical oxidation of glucose on Pt-based catalysts surfaces in basic electrolyte, and the potential was vs RHE.

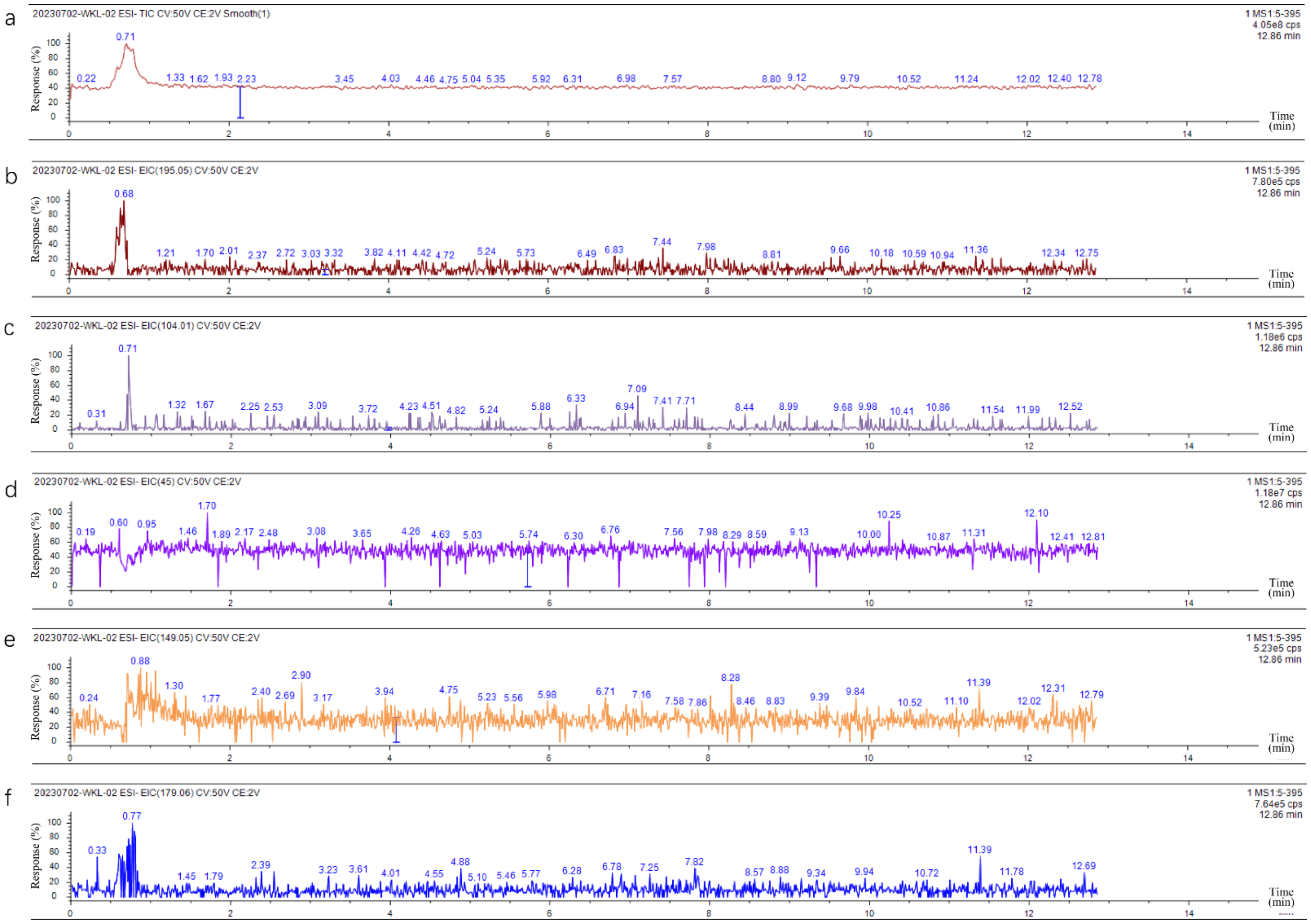

Figure S14. (a) Total ion current chromatogram of the electrolyte for GOR performed on PtPdCu NWs catalyst. (b) Gluconic acid ion current chromatogram. (c) Glucaric acid ion current chromatogram. (d) Formic acid ion current chromatogram. (e) Arabinose ion current chromatogram. (f) Glucose ion current chromatogram.

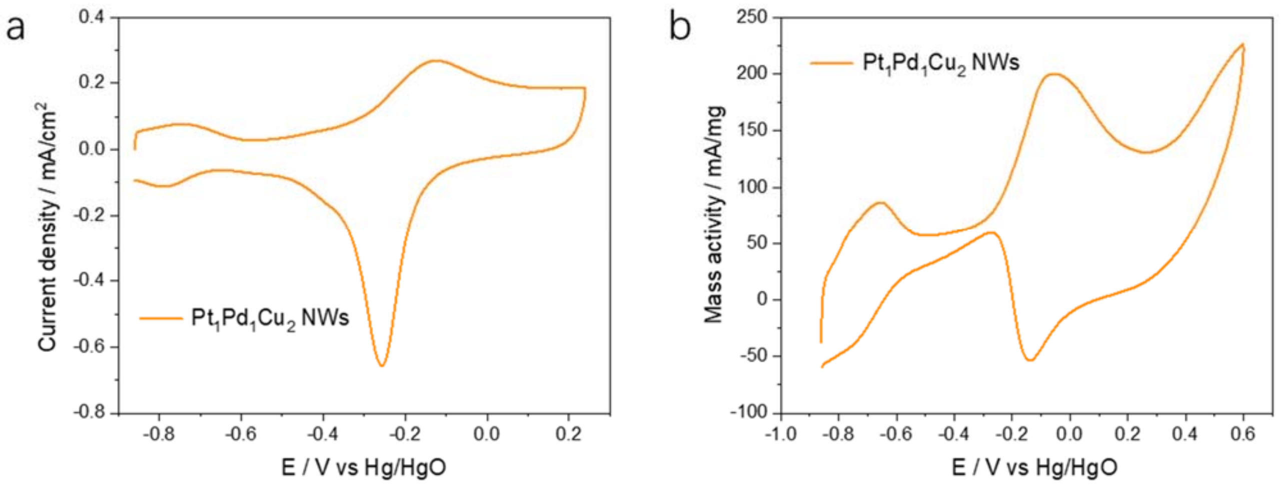

Figure S15. (a) CVs of the $Pt_1Pd_1Cu_2$ NWs in 0.5 M KOH with scan rate of 50 mV/s; (b) CVs in 0.5 M KOH containing 0.1 M glucose with scan rate of 50 mV/s.

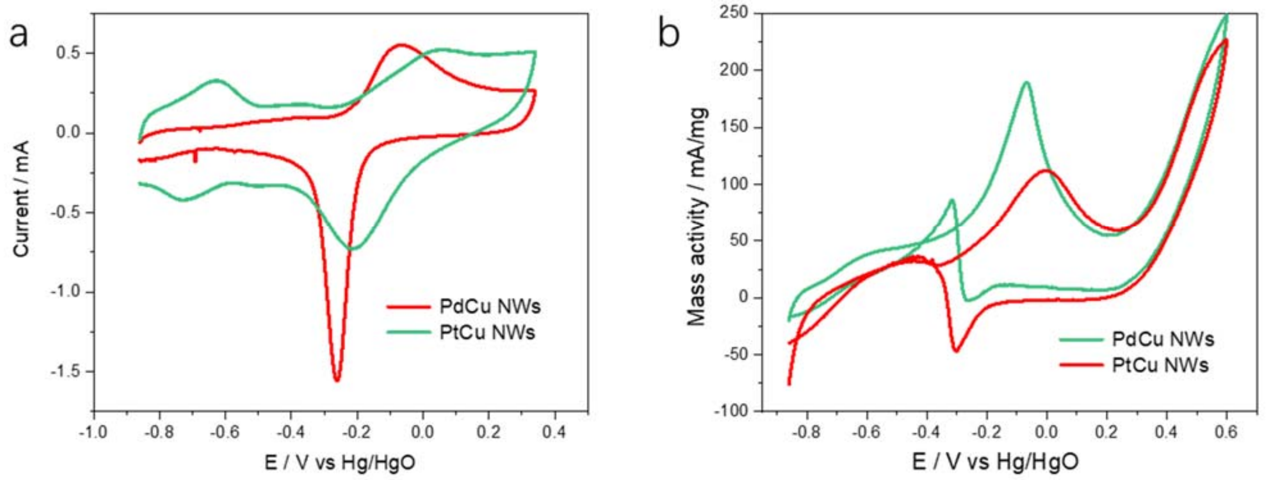

Figure S16. (a) CVs of the PdCu NWs and PtCu NWs in 0.5 M KOH with scan rate of 50 mV/s; (b) CVs in 0.5 M KOH containing 0.1 M glucose with scan rate of 50 mV/s.

Table S1. Comparison of electrocatalytic performance of Pt-based or Pd-based electrocatalysts toward GOR.

Electrocatalysts	Electrolyte	ECSA (m^2/g)	Peak current density (A/mg)	References
Ir@Pt	0.5 M NaOH + 50 mM glucose	135.2	0.128	Catal. Commun. 2015;69:114
Pd_3Sn/Se-C	0.1 M KOH+0.5 M glucose	27.1	~0.18	Electrochim. Acta 244 (2017) 16–25
Pd_3Sn/C	0.1 M KOH+0.5 M glucose	20.1	~0.15	
Au@Pt core-shell mesoporous nanoballs	0.1 M NaOH + 5 mM glucose	19	0.016	J Phys Chem C 2015;119:27529
Au@Pt NBs	0.1 M NaOH+5 mM glucose	19	0.016	J. Phys. Chem. C 2015, 119, 27529–27539
Pt NBs	0.1 M NaOH+5 mM glucose	8	0.009	
Pd-Bi/C	0.5 M NaOH+0.5 M glucose	14	2.08	J. Power Sources 287 (2015) 323e333
AuPtPd	0.1 M NaOH+10 mM glucose	50.8	1.01	Intern. J. Hydrogen Energy 45(2020) 19163-19173
AuPt	0.1 M NaOH+10 mM glucose	99.2	0.74	
PtPd	0.1 M NaOH+10 mM glucose	79.4	0.56	
Pt/C	0.1 M NaOH+10 mM glucose	82.5	0.29	
AuPd/rGO	0.1 M NaOH+10 mM glucose	-	0.037	Electrochim. Acta 2016;212:864.
Pd nanocubes	0.1 M NaOH+5 mM glucose	-	0.018	Electrochim. Acta 2016;211:1024.
AuPt	0.1 M NaOH+10 mM glucose	71.8	0.457	Catal. Sci. Technol. 2017;7:2819.
PtPdCu NWs	0.5 M KOH with 0.1 M glucose	41.6	0.29	This work

Table S2. The details of the EIS fitting for Pt/C and PtPdCu NWs at -0.2 V vs Hg/HgO.

Pt/C		PtPdCu NWs	
Z'/Ω	Z''/Ω	Z'/Ω	Z''/Ω
20.901	0.00477	21.947	0.03085
20.901	0.00577	21.947	0.03734
20.901	0.007	21.947	0.04521
20.901	0.00847	21.947	0.05471
20.901	0.01026	21.947	0.06621
20.901	0.01243	21.948	0.08014
20.901	0.01506	21.948	0.09699
20.901	0.01824	21.948	0.11736
20.901	0.02213	21.949	0.1422
20.901	0.02682	21.949	0.17206
20.901	0.03242	21.95	0.20768
20.901	0.03935	21.951	0.25169
20.901	0.0476	21.953	0.30394
20.901	0.05774	21.954	0.36796
20.901	0.06995	21.957	0.44477
20.901	0.08471	21.96	0.53732
20.901	0.10262	21.965	0.64923
20.902	0.1243	21.971	0.78413
20.902	0.15057	21.978	0.94685
20.902	0.18238	21.989	1.1429
20.902	0.22124	22.002	1.381
20.902	0.26803	22.02	1.6659
20.903	0.32395	22.043	2.0043
20.903	0.39315	22.075	2.42
20.904	0.4755	22.115	2.9107
20.905	0.5766	22.169	3.5079
20.907	0.69572	22.237	4.205
20.909	0.84649	22.331	5.0782
20.912	1.0238	22.451	6.0937
20.916	1.2457	22.612	7.3486
20.922	1.4933	22.805	8.7305
20.93	1.8286	23.086	10.575
20.941	2.1996	23.419	12.583
20.956	2.6818	23.883	15.148
20.974	3.2109	24.424	17.909
20.997	3.8426	25.108	21.141
21.035	4.7868	26.195	25.856

21.073	5.6464	27.24	30.039
21.126	6.8143	28.727	35.576
21.195	8.2218	30.602	42.048
21.282	9.914	32.951	49.573
21.393	11.954	35.886	58.316
21.533	14.408	39.526	68.42
21.71	17.358	44.015	80.05
21.933	20.91	49.527	93.404
22.214	25.176	56.239	108.65
22.567	30.295	64.365	125.98
23.012	36.445	74.17	145.63
23.575	43.856	85.967	167.83
24.282	52.736	99.939	192.65
25.173	63.39	116.25	220.31
26.295	76.173	134.95	251.22
27.707	91.475	156.11	286.04
29.486	109.83	180.22	326.02
31.724	131.8	208.29	372.46
34.536	158.08	242.29	426.63
38.073	189.53	285.19	489.36
42.536	227.06	340.71	560.16
48.081	271.72	413.07	636.95
54.829	325.12	506.42	715.56
63.547	389.5	623.81	789.42
75.664	465.93	764.28	849.35
91.643	554.56	923.28	886.49
108.89	656.21	1091.8	894.17
122.64	776.11	1257.8	870.51
131.2	926.51	1410.6	819.04
140.43	1121.4	1542.1	747.5
162.64	1372	1648.9	665.02
213.13	1686.2	1732	579.65
309.43	2068.1	1794.1	497.52
472.98	2519.5	1839.3	422.23
730.98	3037.1	1871.7	355.38
1117	3608.3	1894.5	297.17
1661.6	4198.4		
2388.2	4755.9		
3295.6	5210.6		
4342.8	5489		

References

1. Naik, K.K.; Gangan, A.; Chakraborty, B.; Nayak, S.K.; Rout, C.S. Enhanced Nonenzymatic Glucose-Sensing Properties of Electrodeposited NiCo(2)O(4)-Pd Nanosheets: Experimental and DFT Investigations. *ACS Appl Mater Interfaces* **2017**, *9*, 23894–23903.
2. Souissi, M.; Sahara, R.; Darvishi, S.; Ahadian, S. Responses to comments on "Ni nanoparticle-decorated reduced graphene oxide for non-enzymatic glucose sensing: An experimental and modeling study [Electrochim. Acta 240 (2017) 388–398]". *Electrochim. Acta* **2019**, *300*, 145–149.
3. Ju, Z.; Zhang, Y.; Zhao, T.; Xiao, W.; Yao, X. Mechanism of Glucose–Fructose Isomerization over Aluminum-Based Catalysts in Methanol Media. *ACS Sustain. Chem. Eng.* **2019**, *7*, 14962–14972.
